# Scale-Up Synthesis and Identification of GLYX-13, a NMDAR Glycine-Site Partial Agonist for the Treatment of Major Depressive Disorder

**DOI:** 10.3390/molecules23050996

**Published:** 2018-04-24

**Authors:** Wenchao Li, Jingjian Liu, Minghua Fan, Zhongtang Li, Yin Chen, Guisen Zhang, Zhuo Huang, Liangren Zhang

**Affiliations:** 1 State Key Laboratory of Natural and Biomimetic Drugs, School of Pharmaceutical Sciences, Peking University, Beijing 100191, China; wenchaoli@bjmu.edu.cn (W.L.); jingjian0828@163.com (J.L.); 15122180567@163.com (M.F.); lizhongtang@bjmu.edu.cn (Z.L.); huangz@hsc.pku.edu.cn (Z.H.); 2 Jiangsu Nhwa Pharmaceutical Co., Ltd., 69 Democratic South Road, Xuzhou 221116, Jiangsu, China; chenyin091400@126.com

**Keywords:** GLYX-13, NMDA receptor, chromatography-free synthesis, crystal structure, whole-cell voltage-clamp

## Abstract

GLYX-13, a NMDAR glycine-site partial agonist, was discovered as a promising antidepressant with rapidly acting effects but no ketamine-like side effects. However, the reported synthetic process route had deficiencies of low yield and the use of unfriendly reagents. Here, we report a scaled-up synthesis of GLYX-13 with an overall yield of 30% on the hectogram scale with a column chromatography-free strategy, where the coupling and deprotection reaction conditions were systematically optimized. Meanwhile, the absolute configuration of precursor compound of GLYX-13 was identified by X-ray single crystal diffraction. Finally, the activity of GLYX-13 was verified in the cortical neurons of mice through whole-cell voltage-clamp technique.

## 1. Introduction

Clinical studies have demonstrated that the *N*-methyl-d-aspartate receptor (NMDAR) antagonist ketamine has rapid antidepressant effects on patients with treatment-resistant depression. This finding suggests that depression can be associated with deficits in the glutamatergic system [1]. NMDAR represents the core of the post-synaptic density (PSD) [2], and plays a critical role in the development of diverse central nervous system (CNS) disorders [3]. Meanwhile, chronic inflammatory disorders that can be modulated by NMDAR are associated with an increased prevalence of MDD [4,5,6]. Recently, several drug candidates which bind with the glycine site of NMDAR have entered clinical trials and presented neuroprotective and anti-depressant effects [7]. GLYX-13, a C-terminal amidated tetrapeptide (Thr-Pro-Pro-ThrNH_2_), is a NMDAR glycine-site partial agonist and a promising drug candidate for the treatment of major depressive disorders (MDD). Enhanced release of brain-derived neurotrophic factor (BDNF) is required for the rapid and sustained antidepressant effects of GLYX-13 [8]. Due to its rapid onset effect, GLYX-13 has been granted breakthrough therapy designation by the Food and Drug Administration (FDA) and it is currently in phase III clinical trials [9,10]. In addition, different to other antidepressants in clinical use, GLYX-13 does not show psychotomimetic side effects [11]. Although there are a number of studies on the bioactivity of GLYX-13 [10,12,13,14], there are limited reports regarding to the synthetic route of GLYX-13. Weiguo et al. have published the first synthetic route of GLYX-13 in a patent [15], however, it has drawbacks of low overall yield, and the use of unfriendly reagents and operating conditions, etc. On the basis of the synthetic route reported by the patent, herein, an improved synthetic route to GLYX-13 was designed by a fragment coupling approach commonly used in peptide synthesis. The procedure, that is suitable for scaled-up synthesis, successfully avoids the drawbacks of the original synthetic route. The three aspects of the reaction conditions were optimized as follows: replacement of unsuitable reagents, improvement of the coupling approach and simplification of the post-processing steps. 

## 2. Results and Discussion

**Figure 1:**
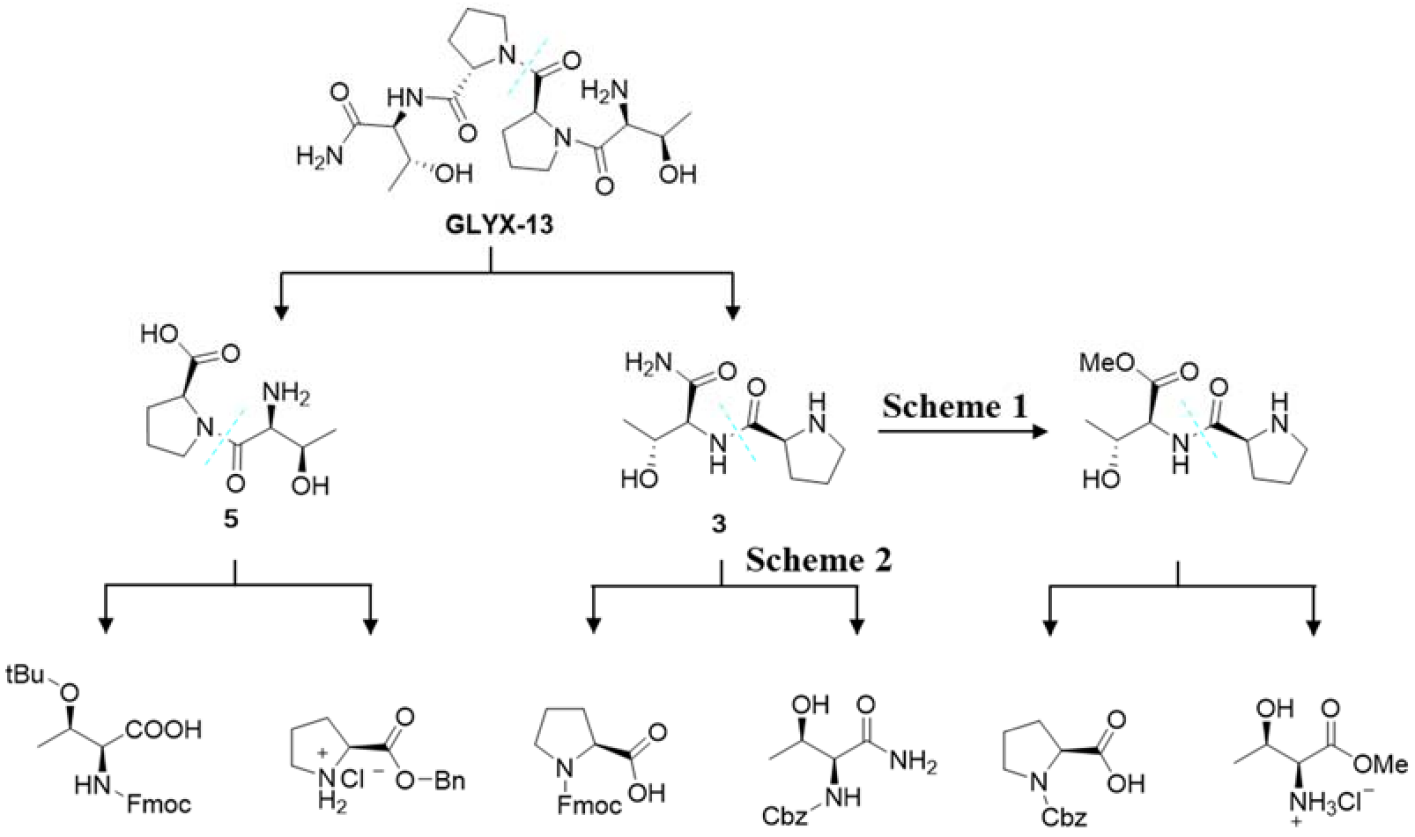
Retrosynthesis of GLYX-13.

GLYX-13 is a tetrapeptide, so its synthesis could be performed by “2 + 2” fragment-coupling strategy (Figure 1). In this approach, *N*-Cbz-l-Thr amide, Fmoc-l-Pro, Fmoc-*O*-^t^Bu-l-Thr and L-Pro benzyl ester hydrochloride was used as starting materials, and intermediates **3** and **5** were firstly synthesized as the key dipeptide segments.

### 2.1. Synthesis of Dipeptide Fragment ***3***

**Scheme 1:**
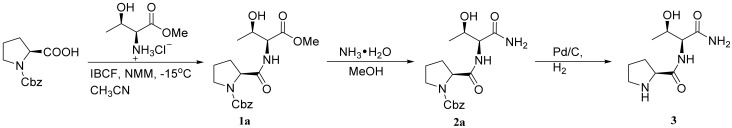
The initial intended synthetic route of intermediate **3**.

Initially, we intended to obtain dipeptide **3** in accordance with the procedure presented in Scheme 1. *N*-Cbz-Pro-OH was employed as starting material to produce the intermediate **3** via three steps including a coupling reaction [16,17,18,19] yielding intermediate **1a** [15], aminolysis of the methyl ester yielding intermediate **2a** [20,21,22] and catalytic hydrogenation yielding **3** (Scheme 1). However, the aminolysis reaction furnished intermediate **2a** in poor yield (40%). Meanwhile, intermediate **3** synthesized via this procedure tended to be a yellowish oily liquid, which was inconvenient for industrial production.

**Scheme 2:**
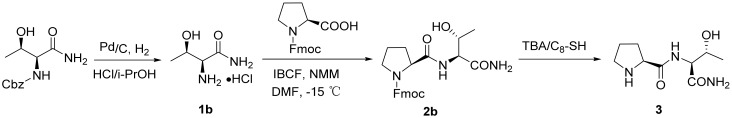
The optimized synthetic route of intermediate **3**.

To improve the synthetic procedure of intermediate **3**, a commercially available compound, *N*-Cbz-Thr amide, was used to generate threonine amide hydrochloride (**1b**) by catalytic hydrogenation. Compound **1b** could be easily purified by the process of acidification and crystallization. Moreover, by crystallization from DMF-water accompanied with the addition of the corresponding seed crystals, intermediate **2b** was obtained in a high purity (Scheme 2).

The reported method to remove the Fmoc group of intermediate **2b** (20% piperidine in DMF) was tedious. After several different deprotection methods were tried, such as AlCl_3_/toluene, DMSO (120 °C), and catalytic hydrogenation [23,24,25,26,27,28,29] *tert*-butyl amine (TBA) was chosen as the optimal deprotection agent accompanied by 1-octanethiol (C_8_-SH) added as an efficient scavenger of dibenzofulvene, a reactive by-product derived from Fmoc removal [24]. TBA is volatile and the by-product was easily removed by washing with ethyl acetate. Finally, intermediate **3** was obtained as white solid by recrystallization from ethyl acetate/ethanol (1:1). In brief, the optimized synthetic route shown in Scheme 2 afforded both high yield and involved easy handling, which were particularly suitable for scaled-up synthesis.

### 2.2. Synthesis of Dipeptide Segment ***5***

**Scheme 3:**
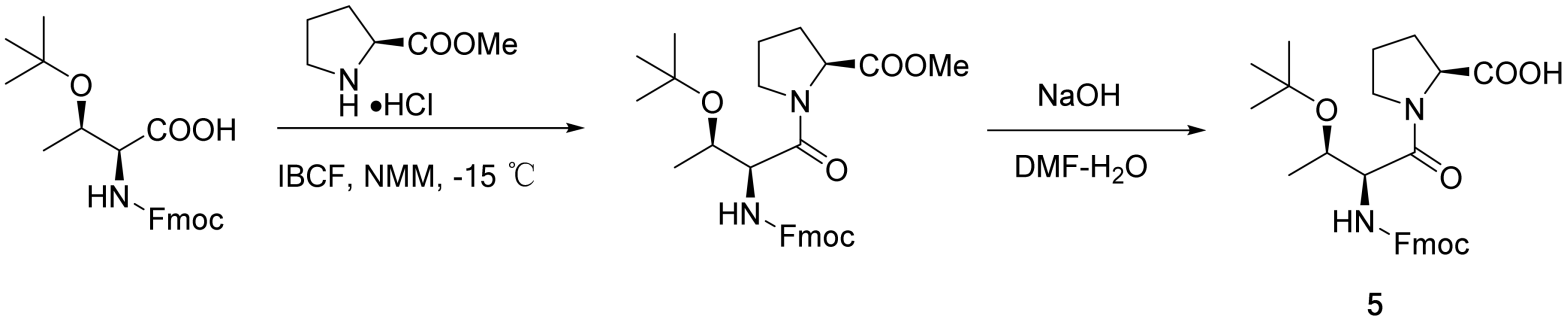
The initial synthetic route to intermediate **5**.

In the synthetic route reported by Weiguo et al., intermediate **5** was synthesized by a two-step reaction including coupling reaction and deprotection of a methyl ester, in which Fmoc-Thr(^t^Bu)-OH and proline methyl ester hydrochloride served as starting materials (Scheme 3). However, the highly hygroscopic proline methyl ester hydrogen chloride and the inefficient coupling reaction mediated by isobutyl chloroformate (IBCF) led to a low yield of the desired product. Moreover, the hydrolysis of the methyl ester caused undesired removal of the Fmoc group.

**Scheme 4:**
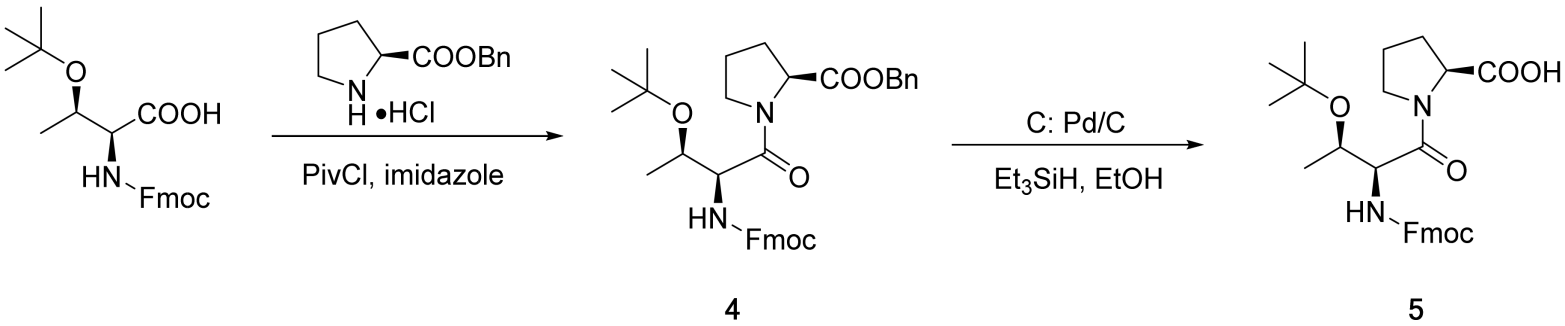
Improved synthetic route of **5**.

To overcome the above problems, a new procedure was designed by us, in which proline benzyl ester hydrochloride is not highly hygroscopic and IBCF was replaced by pivaloyl chloride (PivCl) (Scheme 4). In the following coupling reaction, imidazole was found to be a cost-efficient coupling agent accompanied with minimal impurities as detected by TLC [30,31,32,33,34,35,36,37,38,39,40,41,42]. During the coupling condition optimization in the synthesis of **4**, the combination of EDCI with coupling additives, such as HOBt, DMAP, HOSu and HONp, was also tested but these coupling conditions generated a number of by-products. Similarly, other coupling agents such as TsCl and cyanuric chloride gave **4** in low yield and with a lot of impurities. As for the reaction solvent, the mixture of THF and DCM was chosen due to the excellent solubility of both starting materials (Table 1). 

Because of **4** had a low melting temperature and was difficult to isolate in a solid form, the crude oil liquid of **4** was used in the deprotection step after simple extraction. Removal of the benzyl group of **4** was chemoselective (Table 2) [43,44,45,46], and the impurities were minimized when triethylsilane and ethanol were used as the hydrogen donor and reaction solvent, respectively. 

**Figure 2:**
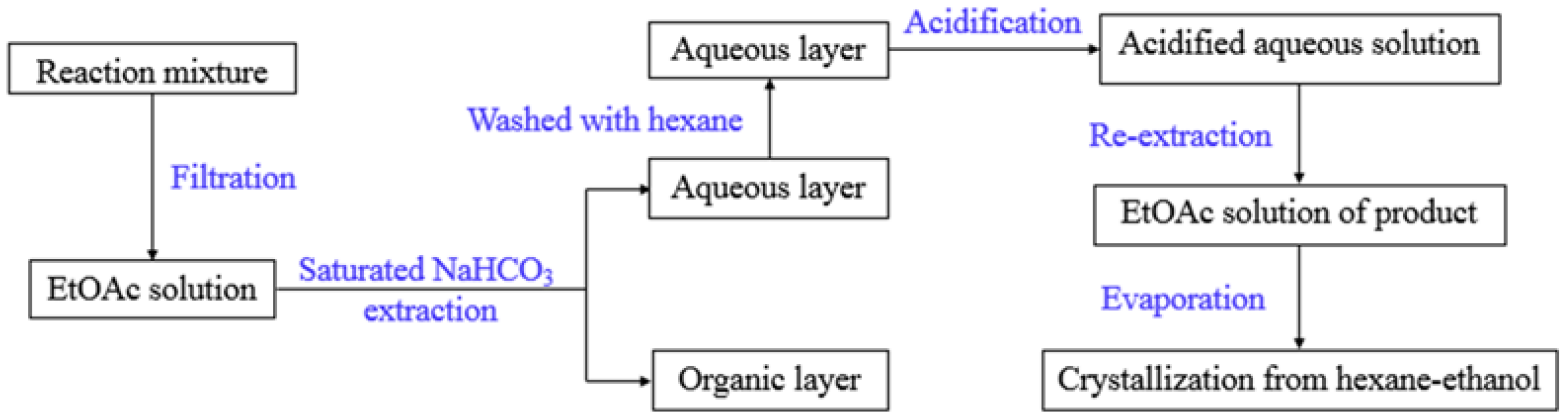
Schematic representation of the isolation of **5** from the reaction mixture.

Isolation of **5** from the reaction mixture was carried out by a combination of neutralized extraction and recrystallization (Figure 2). Partitioning between ethyl acetate and saturated NaHCO_3_ aqueous solution was efficient for elimination of the most organic impurities. Additional hexane washes was needed to ensure that the residual levels of organic impurities in the crude product after neutralized extraction were minimal, and evaporation of ethyl acetate afforded **5** as yellowish oily liquid. Recrystallization from hexane/ethanol (6:1) gave **5** as a white solid.

### 2.3. “2 + 2” Fragment Coupling Reaction and the Preparation of GLYX-13

**Scheme 5:**
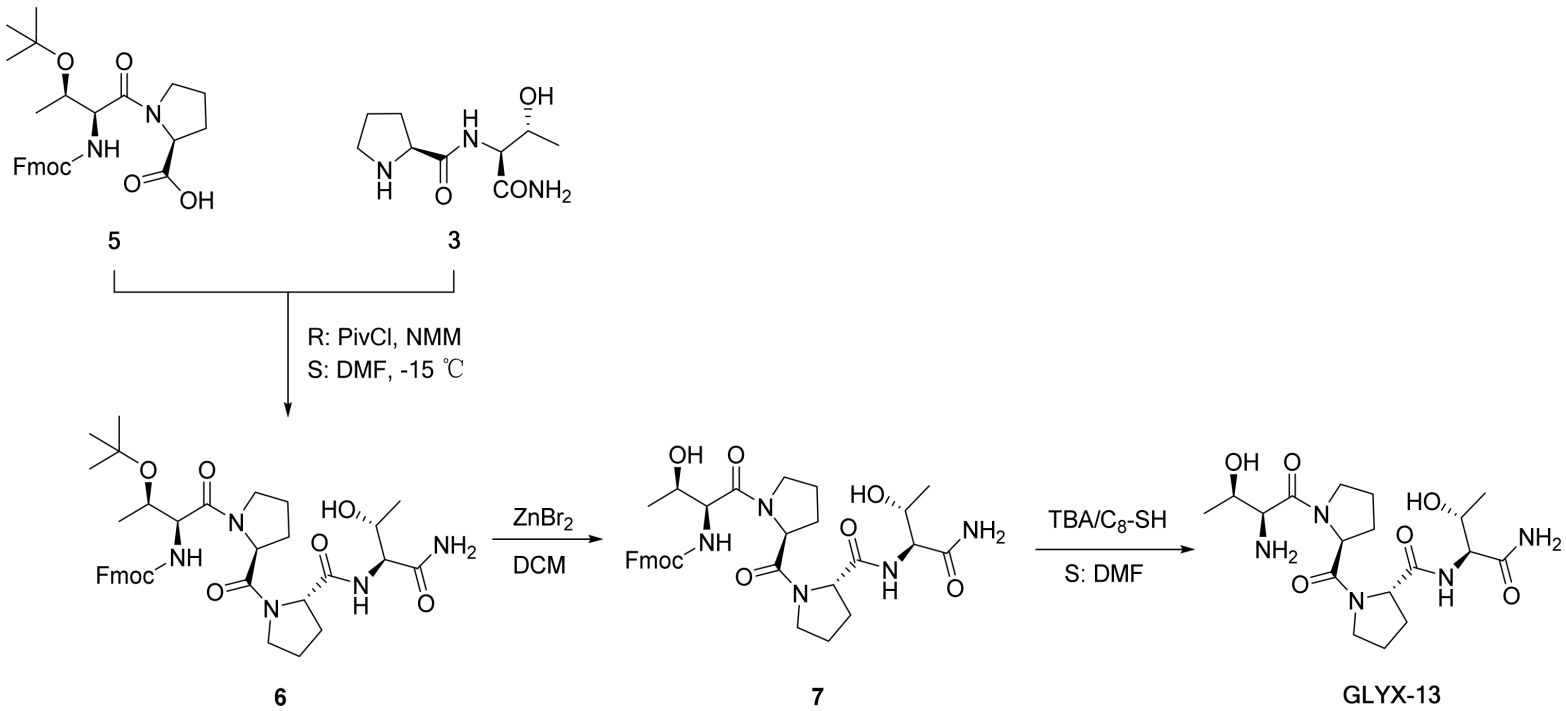
“2 + 2” fragment coupling reaction and final two deprotection reactions.

As we already had success with PivCl as the optimal coupling agent in the synthesis of **4**, PivCl was directly used as coupling agent in the “2 + 2” fragment coupling reaction while *N*-methyl morpholine (NMM) was used as base (Scheme 5). In fact, the coupling reaction of **3** and **5** performed well in these coupling conditions. However, the resulting product was also an oily liquid and inappropriate for purification, so the liquid was directly used in the next step reaction after simple extraction. In the deprotection reaction, the *tert*-butyl ether of **6** was removed using TFA as deprotecting reagent in the original synthetic route. In order to replace TFA with a less corrosive reagent, several deprotection agents were tried, such as 85% phosphoric acid solution, zinc bromide and sodium iodide/cerium chloride [47,48,49]. Finally, zinc bromide was chosen as the optimal reagent for its low cost, environmentally-friendly properties and convenient post-processing. 

In addition, isolation of **7** from the reaction mixture was fairly simple, i.e., slow addition of water into the reaction mixture while stirring. Then, **7** was crystallized as a white solid with a high purity. Finally, the deprotection of **7** was similar to that of **3**, and the work-up also used the same procedures, to afford GLYX-13 as a hygroscopic white solid.

### 2.4. X-ray Single Crystal Diffraction and Powder Diffraction of ***7***

**Figure 3:**
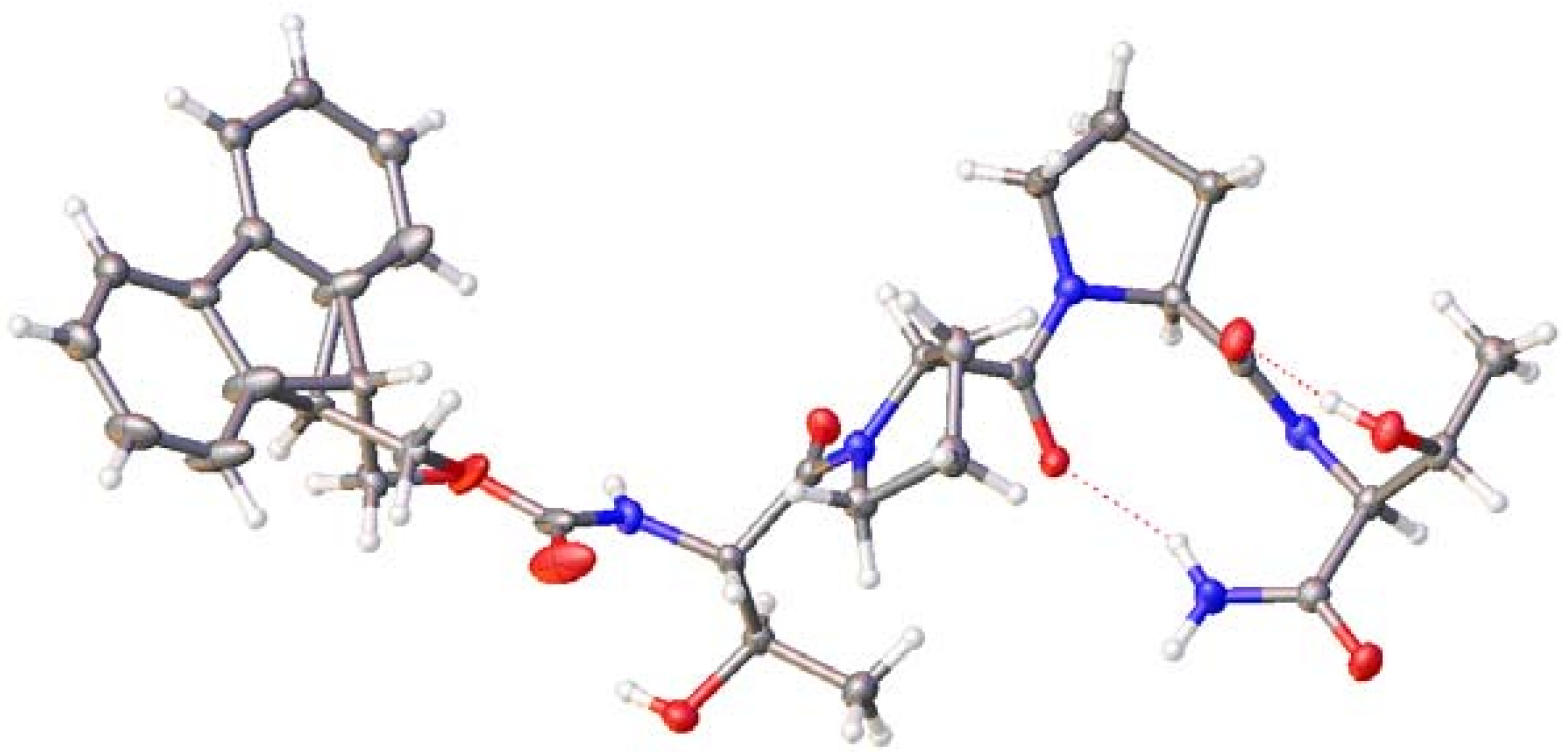
Crystal structure of **7**.

**Figure 4:**
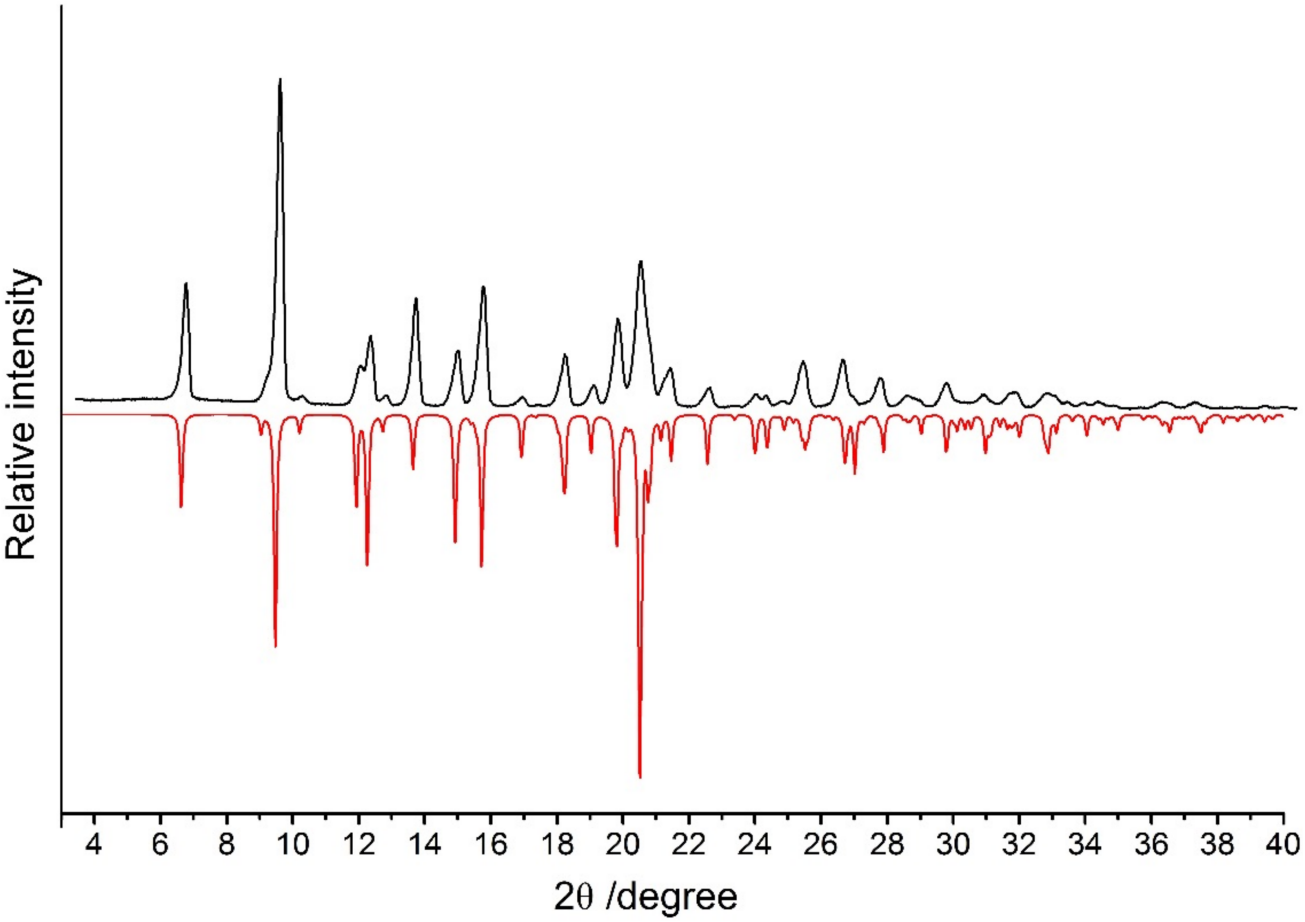
X-ray powder diffraction analysis of **7**. Black represented the tested diffraction pattern diagram of intermediate **7**, red represented the simulated diffraction pattern using Mercury 3.9 software.

GLYX-13 contains six chiral centers, and our plan was to resolve its absolute configuration by X-ray crystal diffraction, however, GLYX-13 proved hard to crystallize although various solvents were tried. We found that **7**, which had the same absolute configuration as GLYX-13, formed crystals much easier than GLYX-13, and the X-ray crystal diffraction showed the right absolute configuration. (Figure 3 and Table S1). Furthermore, a simulated diagram was generated from the single crystal diffraction data by Mercury software. The good match of the experimental diagram with the simulated diagram demonstrated the optical purity of **7** (Figure 4).

### 2.5. Verification the Activity of GLYX-13 on Cortical Neurons

**Figure 5:**
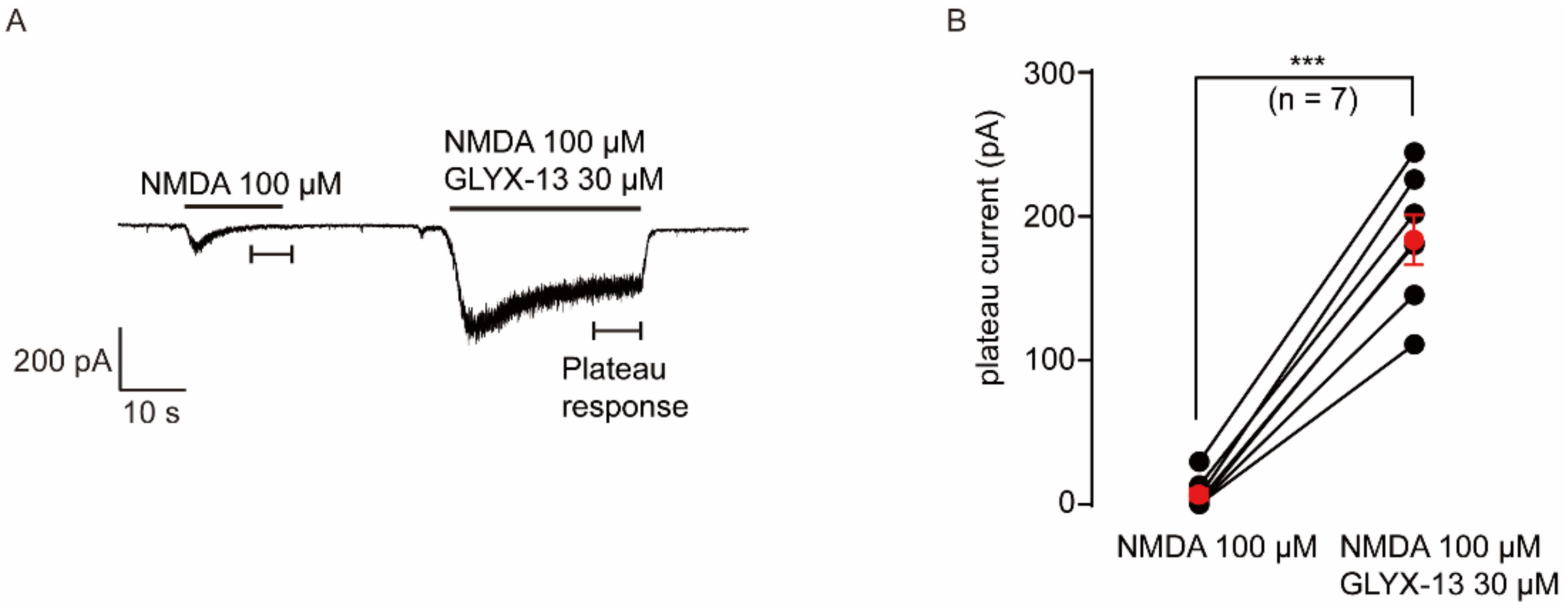

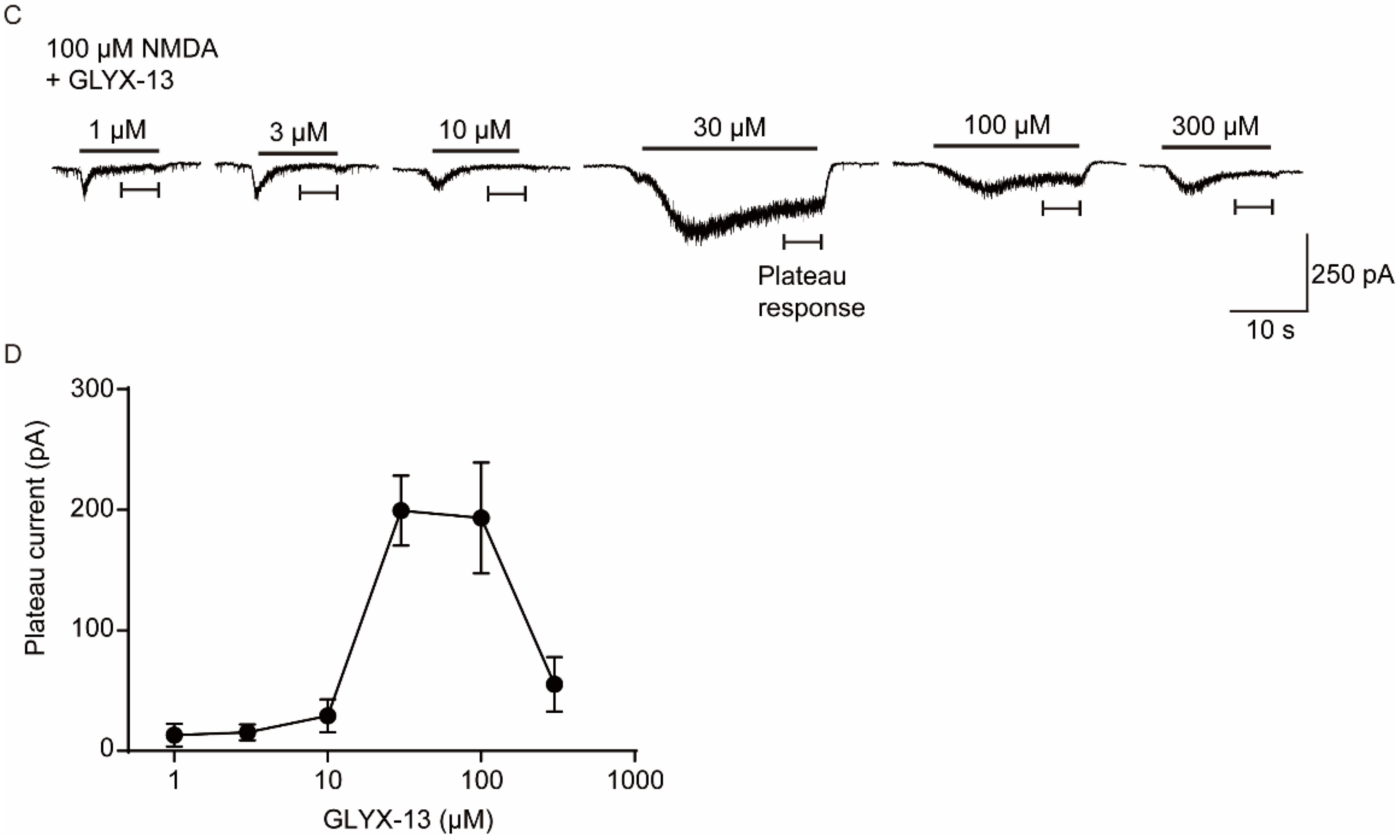
GLYX-13 potentiated responses of NMDA receptors in mice primary cortical neurons. (**A**) Inward *I*_NMDA_ were activated by application of 100 μM NMDA and co-application of 100 μM NMDA and 30 μM GLYX-13; (**B**) Mean and standard error (shown in red) of whole cell *I*_NMDA_ in the presence of 100 μM NMDA and 100 μM NMDA together with 30 μM GLYX-13. Black circles illustrated *I*_NMDA_ from individual experiments. *** *p* < 0.001, paired two-tailed Student’s *t*-test; (**C**) Inward NMDAR-mediated currents were activated by application of 100 μM NMDA with six different concentrations of GLYX-13; (**D**) Concentration-response curve for the effect of GLYX-13 on NMDA receptors. Data were represented as mean ± s.e.m.

To investigate the effect of GLYX-13 on native NMDA receptors, we performed whole-cell voltage-clamp recordings in mice primary cortical neurons to measure NMDAR-mediated currents (*I*_NMDA_) in the presence of 100 μM NMDA without or with 30 μM GLYX-13. The results indicated that 100 μM NMDA alone induced an *I*_NMDA_ with a mean amplitude of 7.26 ± 4.14 pA (*n* = 7), however, co-application of 100 μM NMDA and 30 μM GLYX-13 significantly increased the plateau responses of *I*_NMDA_ to 184.32 ± 17.26 pA (*n* = 7) (Figure 5A,B). To quantitatively understand the activation effect of GLYX-13 on *I*_NMDA_, we examined the concentration-dependence of *I*_NMDA_ that activated by 100 μM NMDA with different concentrations of GLYX-13 (Figure 5C,D). The results showed that the synthesized GLYX-13 significantly potentiated native NMDA receptors in a dose-dependent manner, and higher concentration of GLYX-13 on *I*_NMDA_ performed a decrease trend. GLYX-13 potentiated plateau response of *I*_NMDA_ in a dose-dependent manner which was consistent with previous reported findings [50].

## 3. Materials and Methods

### 3.1. General Information

TLC was performed on silica gel GF254 plates (Qingdao Haiyang Chemical Co., Ltd., Qingdao, China). Compounds were visualized by irradiation with UV light or by treatment with 0.05 g/mL ninhydrin in ethanol or potassium iodide reagent. ^1^H-NMR and ^13^C-NMR were recorded on a Bruker Avance-400 MHz instrument (Bruker BioSpin AG, Fällanden, Switzerland). HRMS were obtained with a LC-ESI-Q-TOF-MS apparatus (Waters, Milford, MA, USA). HPLC analysis of GLYX-13 was performed on an Agilent LC 1100 system (Agilent Technologies Inc., Santa Clara, CA, USA) equipped with a diode array detector. Chiral analysis of intermediate were carried out using a Shimadzu LC 20AD system (SIMADZU, Kyoto, Japan) with a SPD-20A UV detector or an Agilent LC 1100 system equipped with a diode array detector. Single crystal diffraction analysis of **7** was performed on a MicroMax-003 (Rigaku Corporation, Tokyo, Japan) X-ray single crystal diffractometer and powder diffraction analysis of **7** was performed by a Bruker D8 FOCUS powder diffractometer (Bruker BioSpin AG, Fällanden, Switzerland). The L-amino acids (protected or free) were obtained from GL Biochem Ltd. (Shanghai, China). Other reagents were provided by Aladdin (Shanghai, China). The organic solvents were commercially available products (Lingfeng Chemical Reagent Co., Ltd., Shanghai, China) and were used without further purification.

### 3.2. Synthesis

#### 3.2.1. Thr-NH_2_·HCl (**1b**)

*N*-Cbz-ThrNH_2_ (504 g, 2 mol) was dissolved in methanol, followed by addition of 10% of Pd/C (50.4 g, 10 wt %), and the resulting suspension was stirred at room temperature under hydrogen (1.0 MPa) for 12 h. Pd/C was filtered and the filtrate was evaporated in vacuum to furnish the crude product as yellowish oily liquid, which was redissolved in 3 L of acetone, followed by slow addition of a solution of HCl in iPrOH (500 mL) at 0–5 °C under stirring. The precipitated white solid was filtered and dried under high vacuum (45 °C, 0.1 MPa) to afford **1b** 289 g (93.5% yield). ^1^H-NMR (400 MHz, D_2_O) δ 4.15 (p, *J* = 6.1 Hz, 1H), 3.86 (d, *J* = 5.2 Hz, 1H), 1.25 (d, *J* = 6.5 Hz, 3H). ^13^C-NMR (101 MHz, D_2_O) δ 170.25, 66.04, 58.40, 18.80. HRMS (ESI-TOF+) *m*/*z* ([M + H]^+^), calcd. for C_4_H_11_N_2_O_2_ + H^+^: 119.0815, found 119.0817.

#### 3.2.2. Fmoc-Pro-ThrNH_2_ (**2b**)

A four-necked flask was charged with a solution of Fmoc-Pro-OH (337 g, 1 mol) in DMF containing NMM (101 g, 1 mol) under nitrogen, which was cooled to −20 °C, IBCF was added dropwise to the reaction vessel while maintaining the reaction mixture below −15 °C. **1** was added as solids in one portion to the reaction mixture at the same temperature, followed by dropwise addition of NMM (101 g, 1 mol). After the completion of the addition, the reaction mixture was gradually warmed to room temperature. 1 L of water was poured into the reaction mixture under vigorous stirring, followed by addition of the seed crystal resulting in white solid formed, which was then filtered and dried with oven to afford **2** 585 g (89.2% yield). ^1^H-NMR (400 MHz, DMSO-*d*_6_) δ 7.98–7.56 (m, 5H), 7.50–7.29 (m, 4H), 7.14 (m, 2H), 4.86 (dd, *J* = 18.2, 5.3 Hz, 1H), 4.58–4.13 (m, 4H), 4.13–3.94 (m, 2H), 3.56–3.36 (m, 2H), 2.31–1.76 (m, 4H), 1.00 (dd, *J* = 14.9, 6.3 Hz, 3H). ^13^C-NMR (101 MHz, DMSO-*d*_6_) δ 172.64, 172.39, 154.80, 144.38, 141.23, 128.17, 127.67, 125.64, 120.58, 67.17, 66.89, 60.54, 60.42, 58.41, 47.09, 24.46, 23.46, 20.53. HRMS (ESI-TOF+) *m*/*z* ([M + H]^+^), calcd. for C_24_H_27_N_3_O_5_ + H^+^: 438.4960, found 438.2026.

#### 3.2.3. Pro-ThrNH_2_ (**3**)

Compound **2** (437 g, 1 mol) was dissolved in TBA (3 L) containing C_8_-SH (435 mL, 2.5 mol), and the reaction mixture was stirred at room temperature for 30 min. Next 6 L of EtOAc was poured into the reaction mixture under vigorous stirring resulting in the precipitation of product, which was filtered and dried under high vacuum (45 °C, 0.1 MPa). The product was purified by recrystallization from ethyl acetate/EtOH (1:1) to afford **3** 206 g (95.8% yield). ^1^H-NMR (400 MHz, D_2_O) δ 4.24–4.06 (m, 2H), 3.80–3.66 (m, 1H), 2.97–2.73 (m, 2H), 2.07 (s, 1H), 1.68 (d, *J* = 3.2 Hz, 2H), 1.10 (dd, *J* = 6.2, 2.5 Hz, 3H). ^13^C-NMR (101 MHz, D_2_O) δ 177.58, 174.58, 66.97, 59.99, 58.45, 46.45, 30.35, 25.27, 18.78. HRMS (ESI-TOF+) *m*/*z* ([M + H]^+^), calcd. for C_9_H_17_N_3_O_3_ + H^+^: 216.1270, found 216.1342.

#### 3.2.4. Fmoc-Thr(^t^Bu)-Pro-OH (**5**)

A four-necked flask was charged with a solution of Fmoc-Thr(^t^Bu)-OH (397 g, 1 mol) in THF containing imidazole (68 g, 1 mol) under nitrogen, which was cooled to −20 °C, PivCl was added dropwise while maintaining the reaction mixture below −15 °C. Pro-OBzl·HCl was dissolved in DCM containing imidazole (68 g, 1 mol) and then the obtained solution was added dropwise to the reaction mixture at the same temperature. After the completion of the addition, the reaction mixture was allowed to stir at room temperature. After the reaction was finished, the reaction mixture was filtered and the filtrate was evaporated in vacuum to afford the crude product as yellowish oily liquid, which was redissolved in EtOAc and washed with water and saturated NaHCO_3_ solution. The separated organic layer was dried over anhydrous MgSO_4_, filtered and concentrated under vacuum to afford the crude product as an oily liquid, which was dissolved in EtOH, followed by addition of Pd/C and slow addition of Et_3_SiH at 0–10 °C. After the completion of the reaction, the Pd/C was filtered and the filtrate was evaporated in vacuum to furnish the crude product as oily liquid, which was dissolved in EtOAc. The resulting EtOAc solution was extracted with saturated NaHCO_3_ solution, and the combined aqueous solution was washed with n-hexane and acidified with diluted hydrochloride aqueous solution resulting in precipitation of product, which was re-extracted with EtOAc. The obtained EtOAc solution was dried over anhydrous MgSO_4_, filtered and concentrated in vacuum to give the crude product as oily liquid, which was purified by recrystallization from *n*-hexane/EtOH (6:1) to furnish the product as a white solid, which was then dried under vacuum (45 °C, 0.1 MPa) to afford **5** 267.5 g (54% yield). ^1^H-NMR (400 MHz, CDCl_3_) δ 7.79 (d, *J* = 7.5 Hz, 2H), 7.62 (d, *J* = 7.4 Hz, 2H), 7.43 (t, *J* = 7.2 Hz, 2H), 7.33 (dd, *J* = 15.3, 7.8 Hz, 2H), 5.70 (d, *J* = 7.8 Hz, 1H), 4.68 (d, *J* = 7.1 Hz, 1H), 4.56–4.47 (m, 1H), 4.40 (t, *J* = 10.0 Hz, 2H), 4.24 (t, *J* = 6.7 Hz, 1H), 3.97 (dd, *J* = 16.3, 10.5 Hz, 2H), 3.80–3.66 (m, 1H), 2.48–1.77 (m, 5H), 1.33–1.02 (m, 12H). ^13^C-NMR (101 MHz, CDCl_3_) δ 172.88, 171.23, 155.99, 143.90, 141.32, 127.73, 127.06, 125.12, 120.00, 74.92, 68.72, 67.13, 59.76, 57.47, 48.39, 47.18, 28.32, 27.95, 24.92, 19.00. HRMS (ESI-TOF+) *m*/*z* ([M + H]^+^), calcd. for C_28_H_34_N_2_O_6_ + H^+^: 495.2417, found 495.2497.

#### 3.2.5. Fmoc-Thr-Pro-Pro-ThrNH_2_ (**7**)

A four-necked flask was charged with a solution of **5** (247 g, 0.5 mol) in DMF containing NMM (50.5 g, 0.5 mol) under nitrogen, which was cooled to −20 °C, PivCl (60.5 g, 0.5 mol) was added dropwise while maintaining internal temperature below −15 °C. Compound **3** was added as a solid in one portion and then the reaction mixture was allowed to stir at room temperature. After the completion of the reaction, water was poured into the reaction mixture and the resulting mixture was extracted with EtOAc, and the combined organic layers was washed with saturated NaHCO_3_ solution and dried over anhydrous MgSO_4_, filtered and concentrated under vacuum to afford the crude product as an oily liquid, which was dissolved in DCM (500 mL), followed by addition of ZnBr_2_ (562.5 g, 2.5 mol). The resulting reaction mixture was stirred at room temperature for 12 h. Water was added and the reaction mixture was stirred at room temperature resulting in crystallization of **7**, which was filtered and dried with an oven to afford **7** 228.5 g (72% yield). ^1^H-NMR (400 MHz, DMSO-*d*_6_) δ 7.90 (d, *J* = 7.5 Hz, 2H), 7.75 (d, *J* = 7.5 Hz, 2H), 7.38 (m, 6H), 7.09 (d, *J* = 19.0 Hz, 2H), 4.68 (m, 3H), 4.46–3.94 (m, 7H), 3.90–3.51 (m, 5H), 2.22–1.63 (m, 8H), 1.21–0.92 (m, 6H). ^13^C-NMR (101 MHz, DMSO-*d*_6_) δ 172.52, 171.77, 171.28, 169.38, 156.52, 144.26, 141.19, 128.10, 127.53, 125.83, 120.56, 67.22, 66.71, 66.20, 60.15, 59.23, 58.30, 58.06, 47.68, 47.32, 47.12, 29.05, 28.63, 25.04, 24.93, 20.54, 20.10. HRMS (ESI-TOF+) *m*/*z* ([M + H]^+^), calcd. for C_33_H_41_N_5_O_8_ + H^+^: 636.3955, found 636.3024.

#### 3.2.6. Thr-Pro-Pro-ThrNH_2_ (**GLYX-13**)

To a suspension of **7** (31.8 g, 0.05 mol) in DMF (50 mL) was added C_8_-SH (22 mL) and TBA (25 mL), and the resulting mixture was vigorously stirred at room temperature for 30 min. EtOAc (50 mL) was poured into the reaction mixture resulting in precipitation of the crude product, which was filtered and dried under high vacuum (45 °C, 0.1 MPa). The dried crude product was purified by recrystallization from *n*-hexane and EtOH (160 mL, 1:1) to furnish GLYX-13 as a white solid, which was dried in the same manner. Product weight: 20 g (97% yield). ${\left[\alpha \right]}_{D}^{30}$ = −112.8 (*c* = 1, H_2_O). ^1^H-NMR (400 MHz, D_2_O) δ 4.60 (dd, *J* = 17.6, 7.7 Hz, 1H), 4.41 (dd, *J* = 15.4, 9.8 Hz, 1H), 4.25–4.08 (m, 2H), 3.86–3.35 (m, 6H), 2.37–2.14 (m, 2H), 2.07–1.70 (m, 6H), 1.20–0.99 (m, 6H). ^13^C-NMR (101 MHz, D_2_O) δ 174.43, 173.21, 172.56, 172.42, 69.31, 67.00, 60.34, 58.79, 58.67, 57.71, 48.20, 47.80, 29.23, 28.21, 26.71, 24.61, 18.79, 18.51. HRMS (ESI-TOF+) *m*/*z* ([M + H]^+^), calcd. for C_18_H_31_N_5_O_6_ + H^+^: 414.2274, found 414.2121. Analyt. HPLC: tR = 7.00 min (>99%).

### 3.3. Cortical Neuronal Cultures

Cortical neurons were prepared from neonatal (P0) C57BL/6 mice of either sex. Animals were decapitated and their brains were dissected under a stereo micro-scope. Cortex was dissociated and digested with 0.25% trypsin (Life Technologies, Framingham, MA, USA) for 30 min at 37 °C. Following trypsinization, DMEM (Life Technologies) supplemented with 10% FBS (Gemini, Rocklin, CA, USA) and DNase solution (Macklin, Shanghai, China) were added. Cells were subsequently dissociated by gentle trituration using a pipette. Cell suspension was collected and then plated onto poly-D-lysine (Sigma-Aldrich Corporation, St. Louis, MO, USA) coated glass coverslips in sterile plastic dishes. After incubation for 4 h at 37 °C in a humidified 95% air/5% CO_2_ atmosphere, the medium was changed with Neurobasal Medium supplemented with 2% B27 and 0.5 mM GlutaMAX-1 (Life Technologies). 

### 3.4. Whole-Cell Voltage-Clamp Recordings

Cortical neurons were used for electrophysiological experiments after 7–14 days in vitro. Cultures were transferred to a glass-bottomed recording chamber perfused with the external solution, containing 142 mM NaCl, 2.5 mM KCl, 2 mM CaCl_2_, 10 mM HEPES, 10 mM Glucose (pH 7.3 with NaOH, 325 mOsm). Pipettes had resistances of 5–8 MΩ when filled with internal pipette solution, containing 122 mM CsCl, 1 mM CaCl_2_, 5 mM MgCl_2_, 10 mM EGTA, 10 mM HEPES, 4 mM Na_2_ATP, 0.3 mM Tris-GTP, 14 mM Tris-phosphocreatinin (pH 7.3 with CsOH, 318 mOsm). Whole-cell current recordings were made from cultured neurons at a holding potential of −70 mV using a HEKA EPC10 amplifier with the PatchMaster.v2.15 (HEKA, Welwyn Garden, United Kingdom). Series resistance was in the order of 10–30 MΩ and was approximately 80% compensated. All recordings were performed at room temperature.

Drugs were diluted from concentrated stock solutions into a modified version of the external solution including 1 μM strychnine and 500 nM TTX. Solution switching was achieved with a rapid solution changer (ALA-VW8, New York, NY, USA). 

Values of plateau response of NMDA-mediated currents obtained from whole-cell voltage-clamp recordings were calculated and analyzed. Group data were represented as mean ± s.e.m. Comparisons between two groups were made using Student’s paired. Statistical significance of differences at *p* < 0.05 is indicated as asterisk (*), *p* < 0.01 is indicated as two asterisk (**) and *p* < 0.001 is shown with three asterisk (***) in all figures.

## 4. Conclusions

A column chromatography-free solution-phase synthetic route of GLYX-13 has been developed with an optimized coupling strategy. Compared with the original route reported in a patent, the overall yield was significantly improved (from 10–15% to 30%), and the work-up procedures were suitable for industrial production. Moreover, the absolute configuration of precursor compound of GLYX-13 was identified by X-ray single crystal diffraction, and the activity of GLYX-13 was verified in the cortical neurons. In summary, all the results indicated that the improved synthetic route of GLYX-13 was economic and scalable and provided the product with good yield and high optical purity.

## Figures and Tables

**Table 1:** Coupling reaction conditions optimization in the synthesis of **4**.

Entry	Method	Base	Reaction Solvent ^a^	Impurity ^b^
1	EDCI/HOBt	DIPEA	DCM	Obvious
2	EDCI/DMAP	DIPEA	DCM	Obvious
3	EDCI/HOSu	DIPEA NaHCO_3_	DCM/THF	Obvious
4	EDCI/HONp	DIPEA NaHCO_3_	DCM/THF	Obvious
5	TsCl	NMM	DCM, THF, ACN	Obvious
6	TsCl	NMI	DCM, THF, ACN	Obvious
7	Cyanuric chloride	NMM	ACN/Acetone/DMF	Obvious
8	Cyanuric chloride	NMM	THF/toluene	Trace
9	PivCl	TEA	THF/DCM	Trace
10	PivCl	Imidazole	THF/DCM	Trace

^a^ THF/DCM was the optimal reaction solvent; ^b^ Impurity was detected by TLC.

**Table 2:** Reaction conditions optimization of benzyl deprotection in the synthesis of **5**.

Entry	Method	Reaction Solvent	Yield (%)
1	Pd/C, H_2_	MeOH	-
2	Pd/C, H_2_	EtOH	-
3	Pd/C, HCOONH_4_	MeOH	-
4	Pd/C, HCOOH	MeOH	-
5	Pd/C, Et_3_SiH	MeOH	65%
6	Pd/C, Et_3_SiH	EtOH	68%

## Supplementary Materials

Supplementary materials are available online. CCDC numbers 1549242 contains the supplementary crystallographic data for this paper. These data can be obtained free of charge via http://www.ccdc.cam.ac.uk/conts/retrieving.html (or from the CCDC, 12 Union Road, Cambridge CB2 1EZ, UK; Fax: +44-1223-336033; E-mail: deposit@ccdc.cam.ac.uk). Electronic Supplementary Materials (ESM) available: Table S1, Figures S1–S21.
