# Supplementary material for: Scale-Up Synthesis and Identification of GLYX-13, a NMDAR Glycine-Site Partial Agonist for the Treatment of Major Depressive Disorder
Source: Molecules. 2018 Apr 24;23(5):996. doi: 10.3390/molecules23050996 (PMC6102568; doi:10.3390/molecules23050996)
Supplement: Supplementary file 1 [file molecules-23-00996-s001.pdf]

Supporting Information

Scale-up synthesis and identification of GLYX-13, a NMDAR glycine-site partial agonist for the treatment of major depressive disorder

Wenchao Li [1, †], Jingjian Liu [1, †], Minghua Fan [1], Zhongtang Li [1], Yin Chen [2], Guisen Zhang [2], Zhuo Huang [1] and Liangren Zhang [1,*]

[1] State Key Laboratory of Natural and Biomimetic Drugs, School of Pharmaceutical Sciences, Peking University, Beijing 100191, P. R. China; wenchaoli@bjmu.edu.cn(W.L.); jingjian0828@163.com(J.L.); 15122180567@163.com(M.F.); lizhongtang@bjmu.edu.cn(Z.L.); huangz@hsc.pku.edu.cn(Z.H.)

[2] Nhwa Institute of Pharmaceutical Research, Xuzhou 221008, P. R. China; chenyin091400@126.com(Y.C.); george_gs@163.com(G.Z.)

* Correspondence: liangren@bjmu.edu.cn; Tel.: +86-10-8280-2567; Fax: +86-10-8280-5063

† Equal contribution.

Table of contents

Table S1. X-ray single crystal diffraction data of 7.

Figure S1. HPLC spectrum of GLYX-13.

Figure S2-S13. NMR spectra of intermediate and GLYX-13.

Figure S14-S19. HRMS spectra of intermediate and GLYX-13.

Figure S20. MS/MS spectra of GLYX-13.

Figure S21. MS/MS fragment ion analysis of GLYX-13.

Table S1 Crystal data and structure refinement for intermediate **7**.

Identification code	GLYX-13-IntVII
Empirical formula	$C_{33}H_{41}N_5O_8$
Formula weight	635.71
Temperature/K	100
Crystal system	monoclinic
Space group	$P2_1$
a/Å	10.23724(8)
b/Å	11.38161(9)
c/Å	13.96504(12)
α/°	90
β/°	107.4386(9)
γ/°	90
Volume/Å^3	1552.37(2)
Z	2
ρ_{calc}g/cm^3	1.360
μ/mm^{-1}	0.810
F(000)	676.0
Crystal size/mm^3	0.43 × 0.16 × 0.12
Radiation	CuKα (λ = 1.54184)
2Θ range for data collection/°	6.634 to 137.58
Index ranges	$-11 \leq h \leq 12$, $-13 \leq k \leq 13$, $-16 \leq l \leq 16$
Reflections collected	59718
Independent reflections	5659 [R_{int} = 0.0448, R_{sigma} = 0.0136]
Data/restraints/parameters	5659/1/446
Goodness-of-fit on F^2	1.033
Final R indexes [I>=2σ (I)]	R_1 = 0.0418, wR_2 = 0.0872
Final R indexes [all data]	R_1 = 0.0420, wR_2 = 0.0874
Largest diff. peak/hole / e Å^{-3}	0.52/-0.55
Flack parameter	-0.05(18)

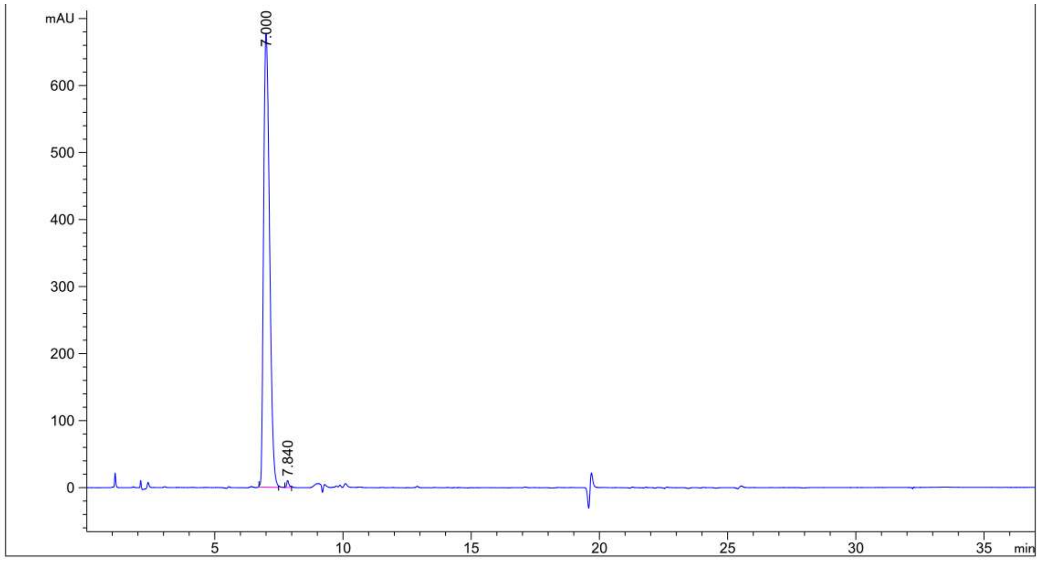

Figure S1. HPLC spectrum of GLYX-13. The HPLC analytic method was described as follows: flow rate: 1 mL/min, reverse C_{18} column (250 mm * 4.6 mm, 5 μm), detection wavelength: 210 nm, Mobile phase A: water (0.1% diethylamine), Mobile phase B: ACN (0.1% diethylamine), gradient elution: 40 min.

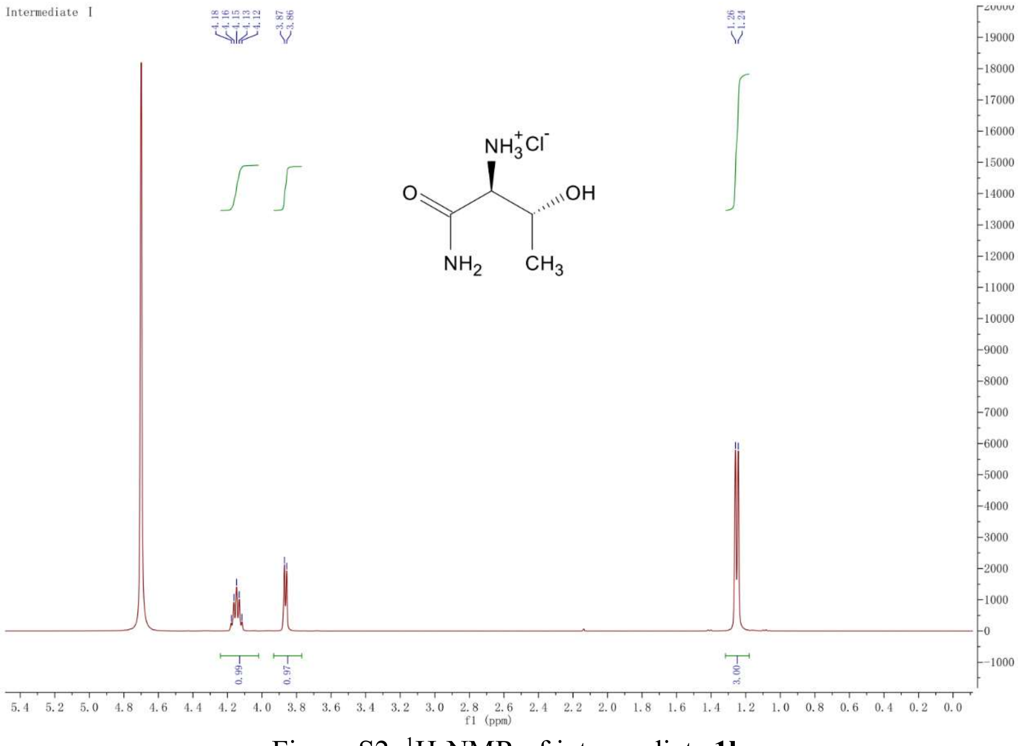

Figure S2. ^{1}H-NMR of intermediate **1b**

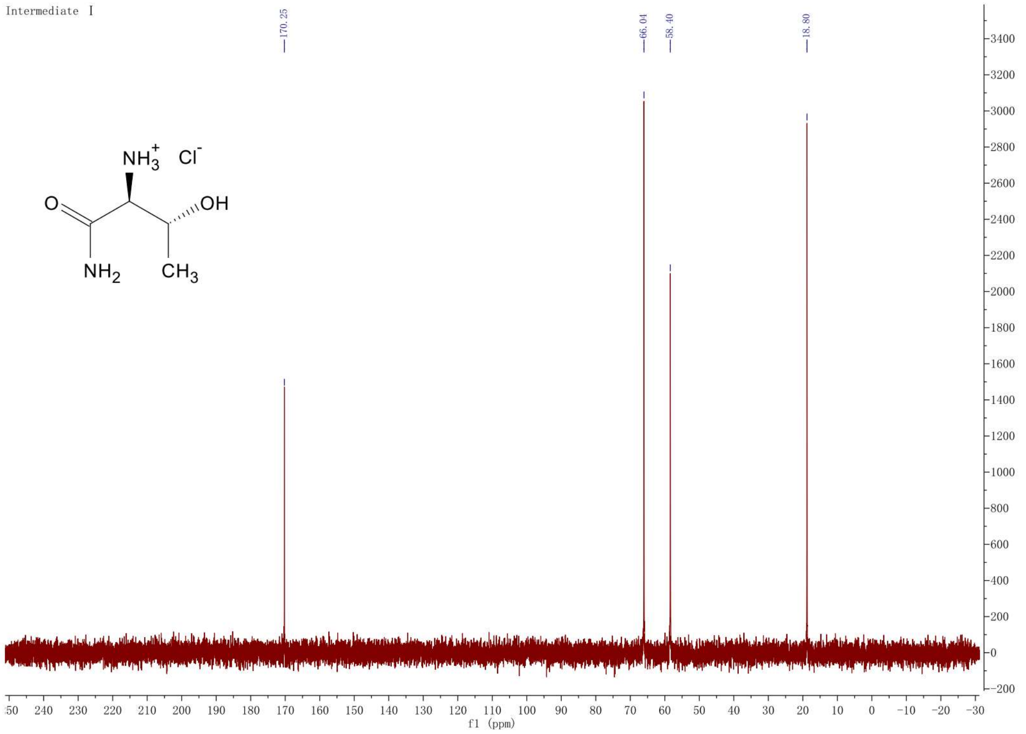

Figure S3. ^{13}C-NMR of intermediate **1b**

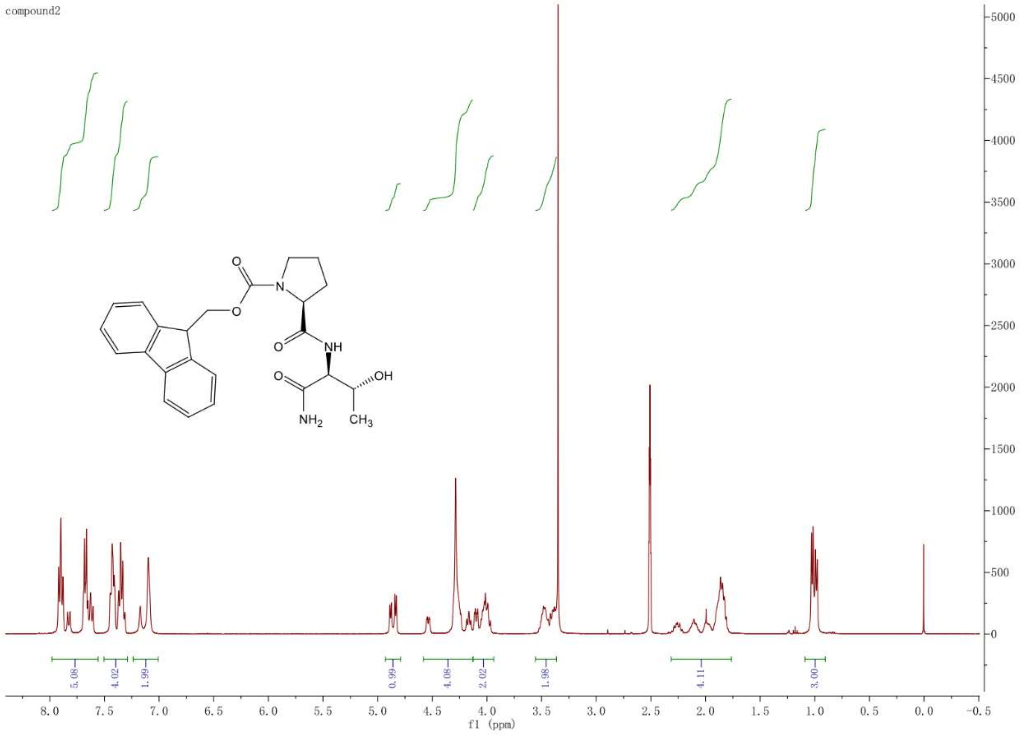

Figure S4. ^{1}H-NMR of intermediate **2b**

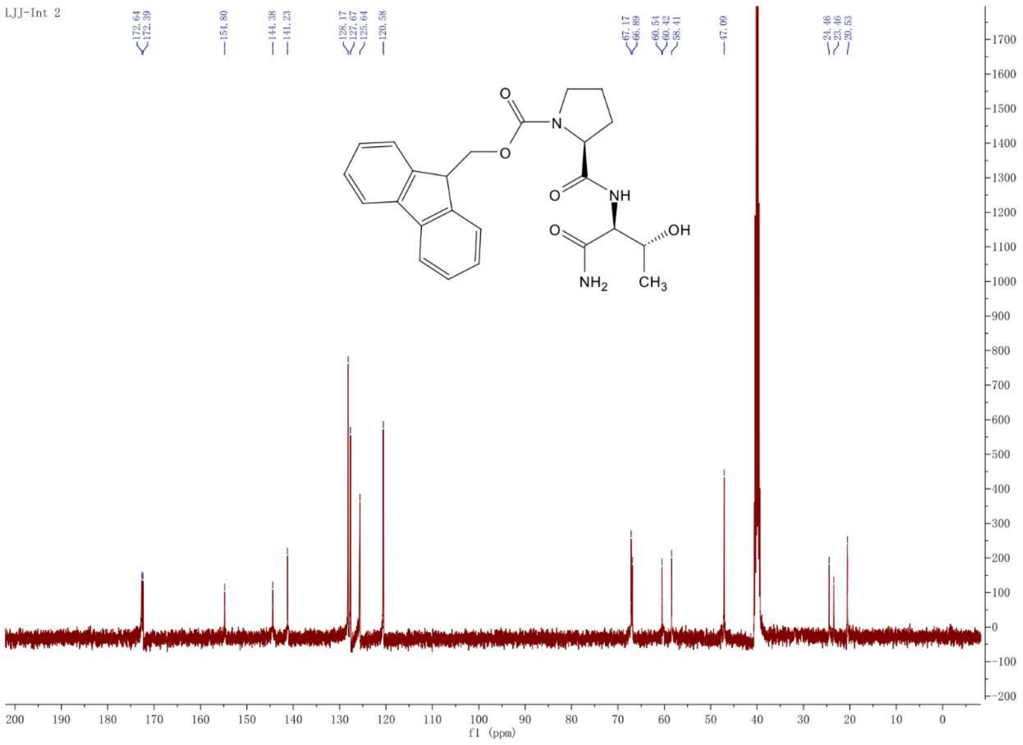

Figure S5. ^{13}C-NMR of intermediate **2b**

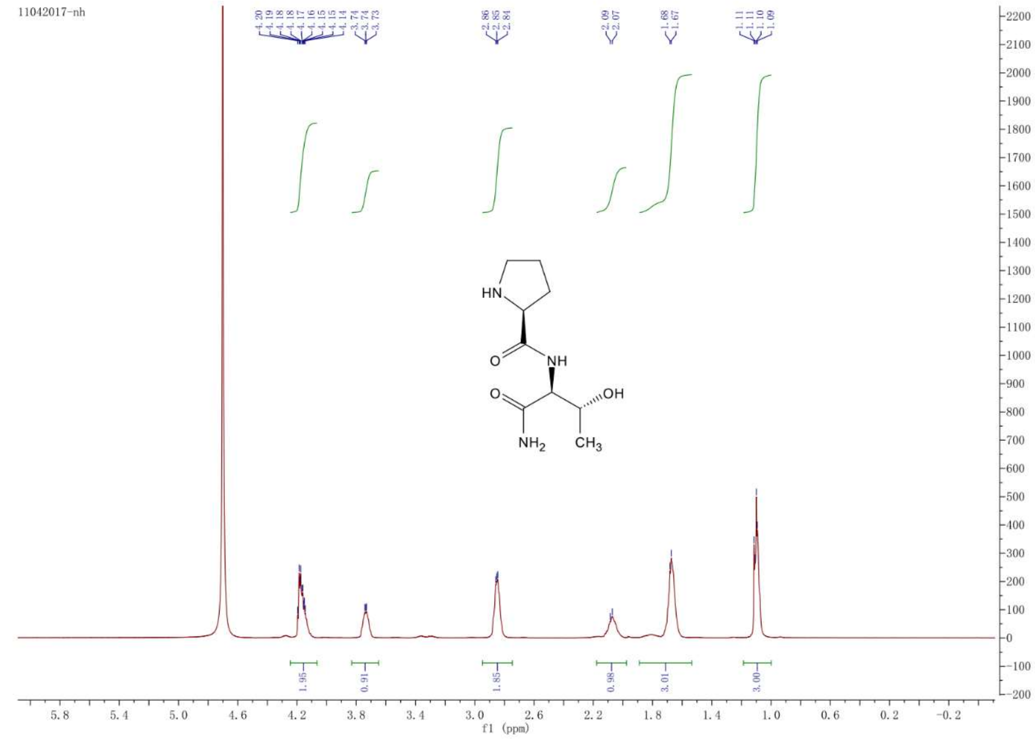

Figure S6. ^{1}H-NMR of intermediate **3**

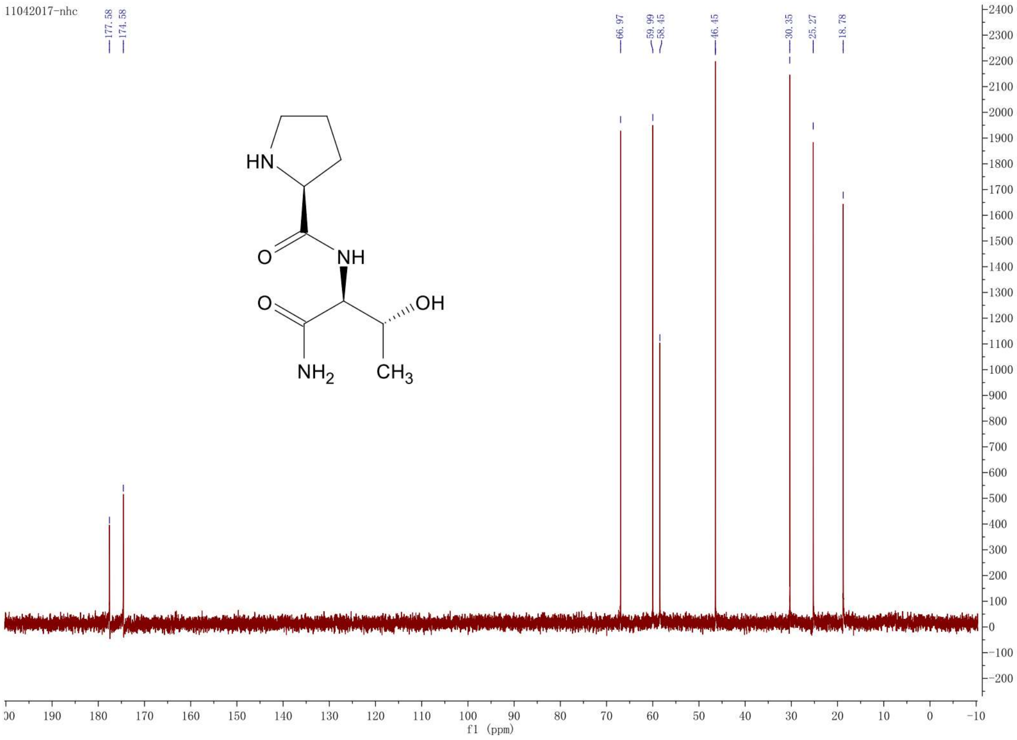

Figure S7. ^{13}C-NMR of intermediate **3**

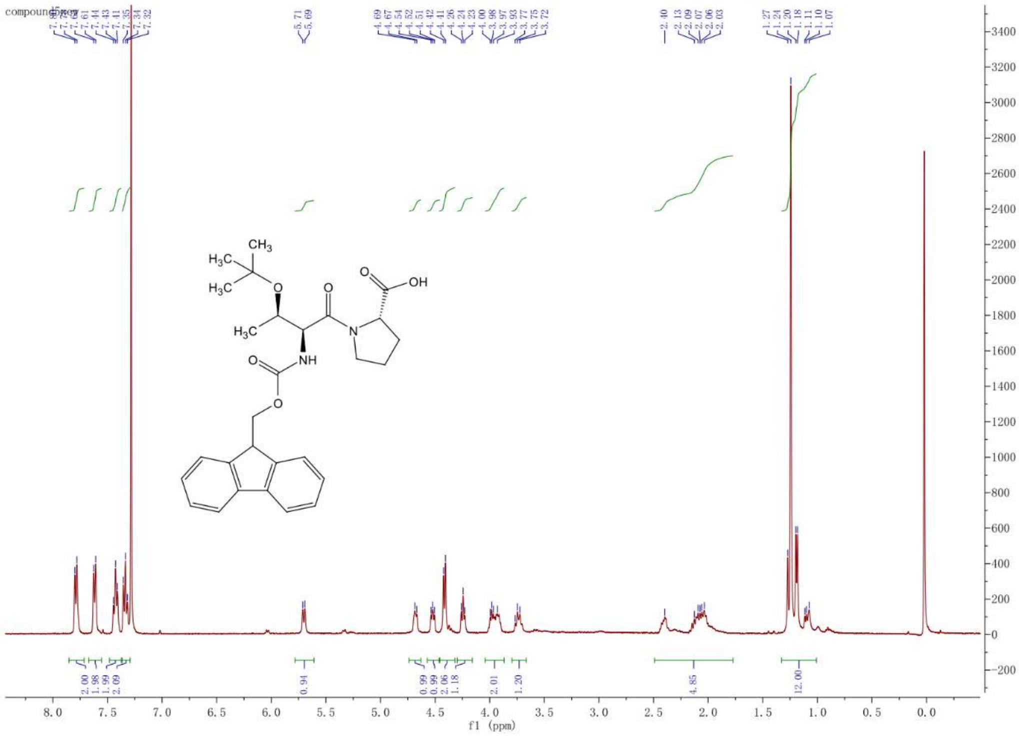

Figure S8. ^{1}H-NMR of intermediate **5**

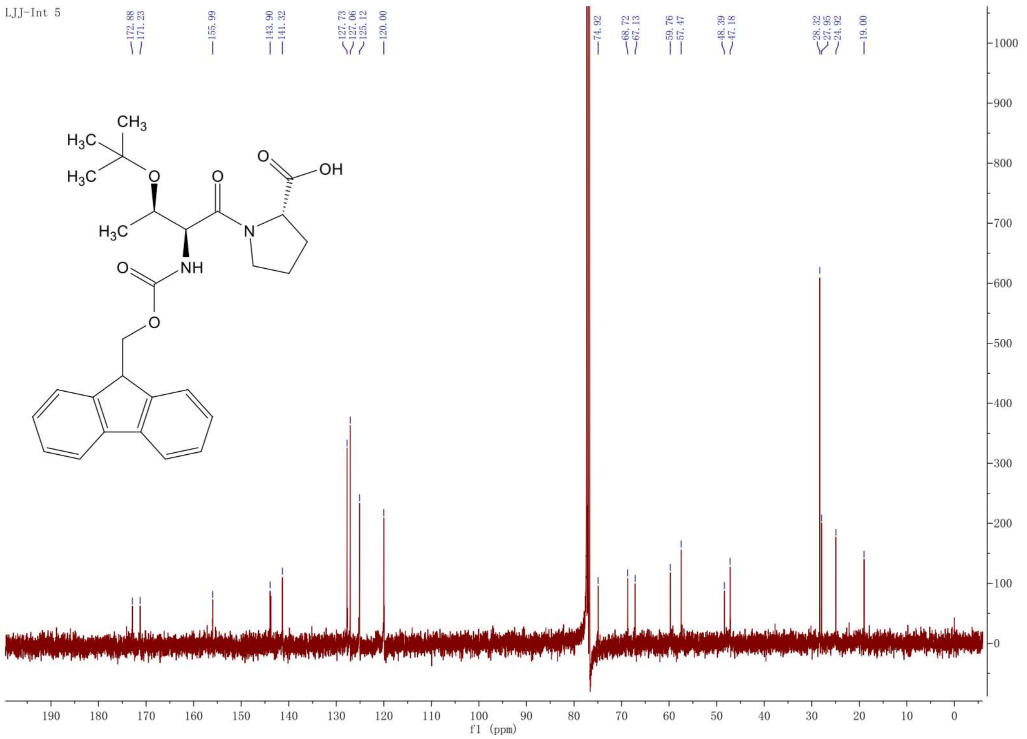

Figure S9. ^{13}C-NMR of intermediate **5**

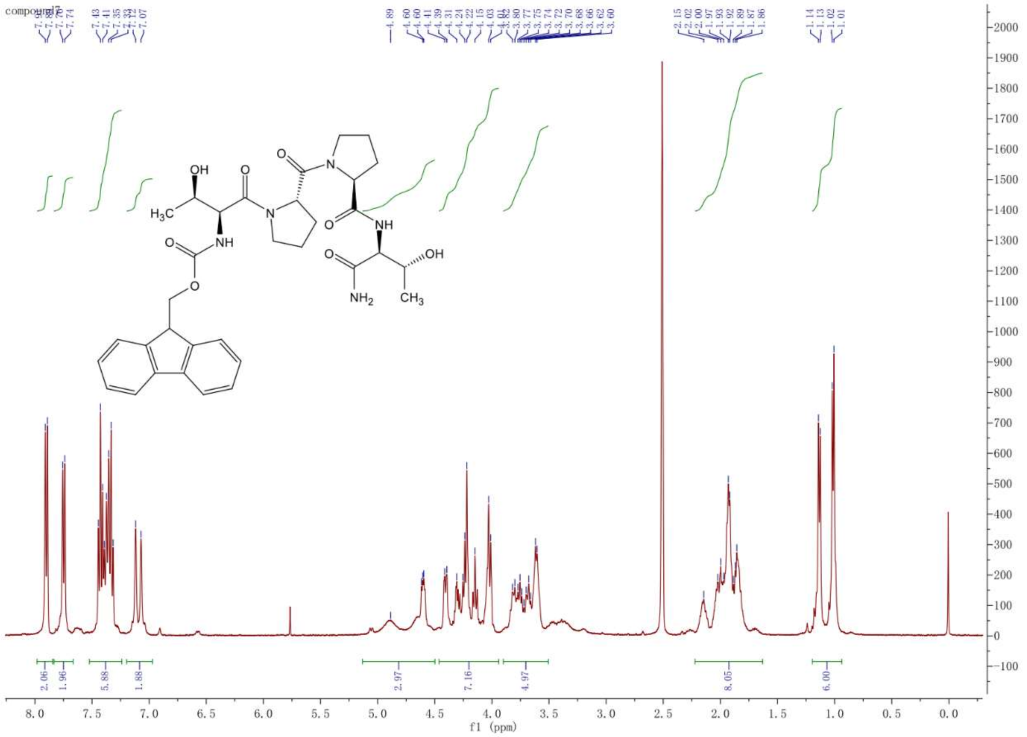

Figure S10. ^{1}H-NMR of intermediate **7**

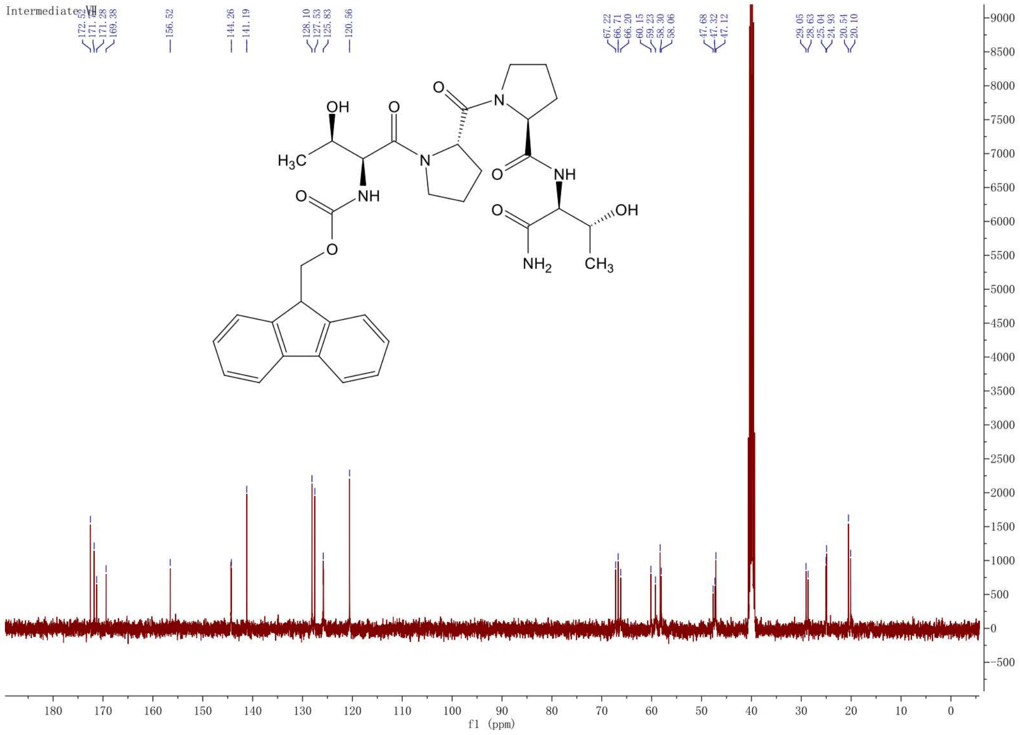

Figure S11. ^{13}C-NMR of intermediate **7**

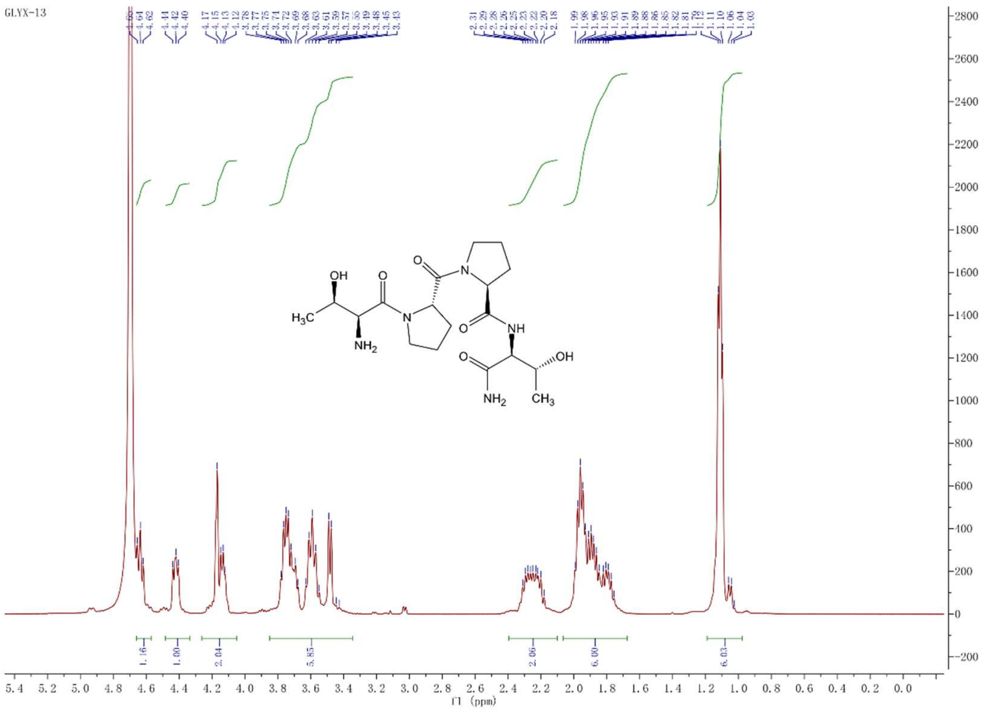

Figure S12. ^{1}H-NMR of GLYX-13

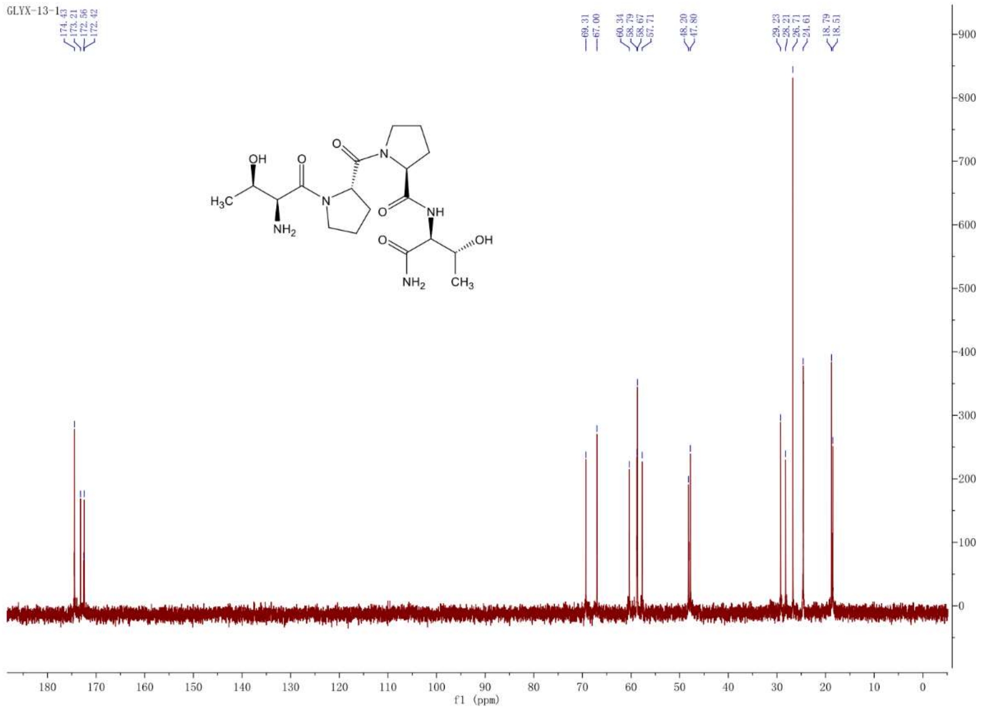

Figure S13. ^{13}C-NMR of GLYX-13

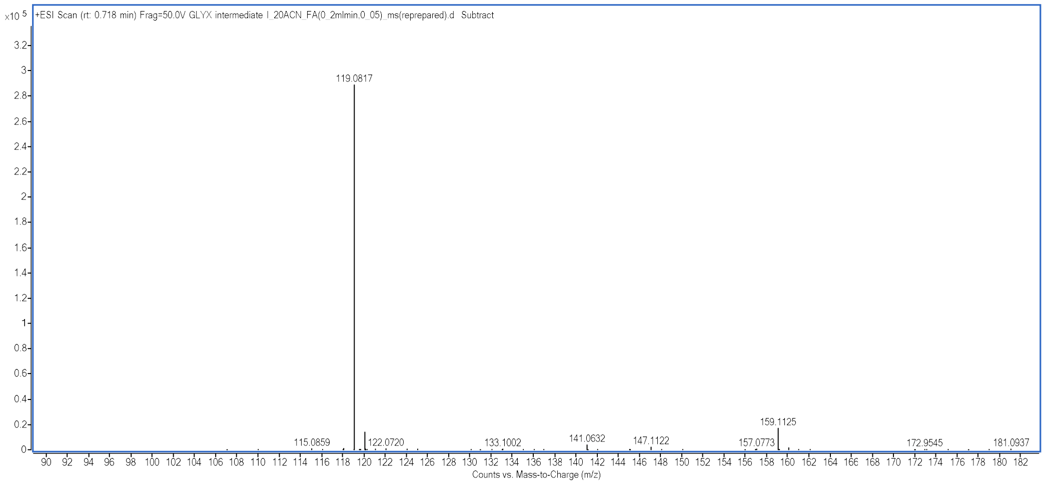

Figure S14. HRMS of intermediate **1b**

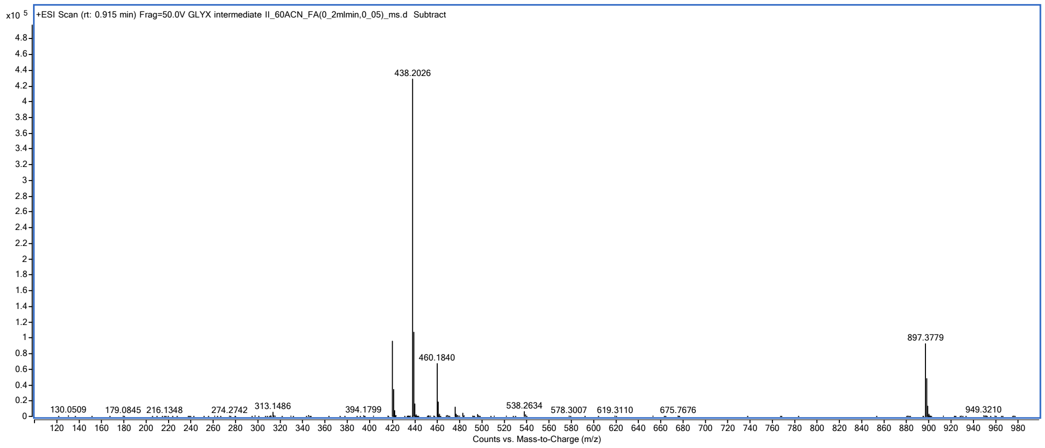

Figure S15. HRMS of intermediate **2b**

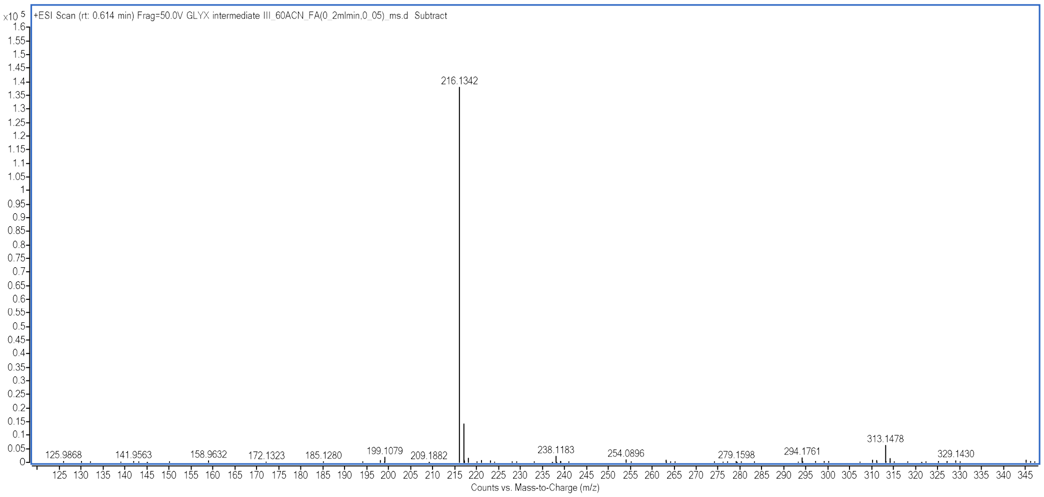

Figure S16. HRMS of intermediate **3**

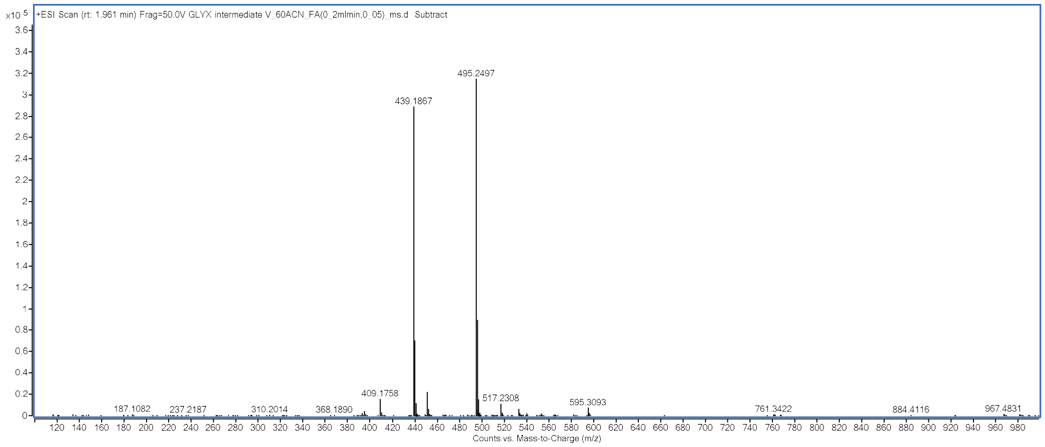

Figure S17. HRMS of intermediate **5**

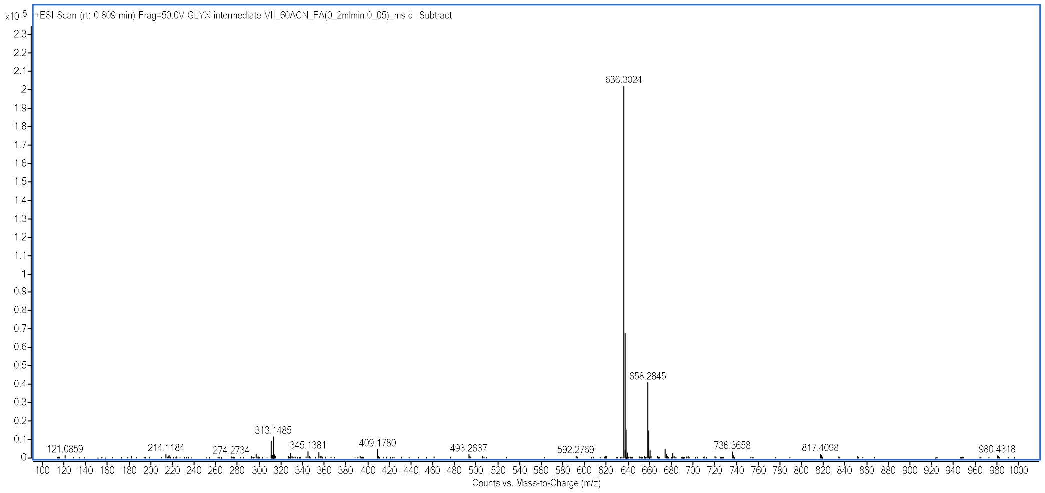

Figure S18. HRMS of intermediate **7**

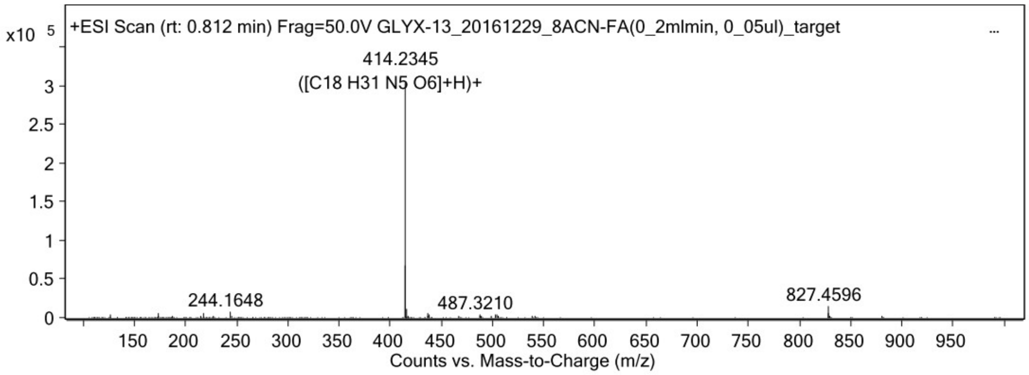

Figure S19. HRMS of GLYX-13

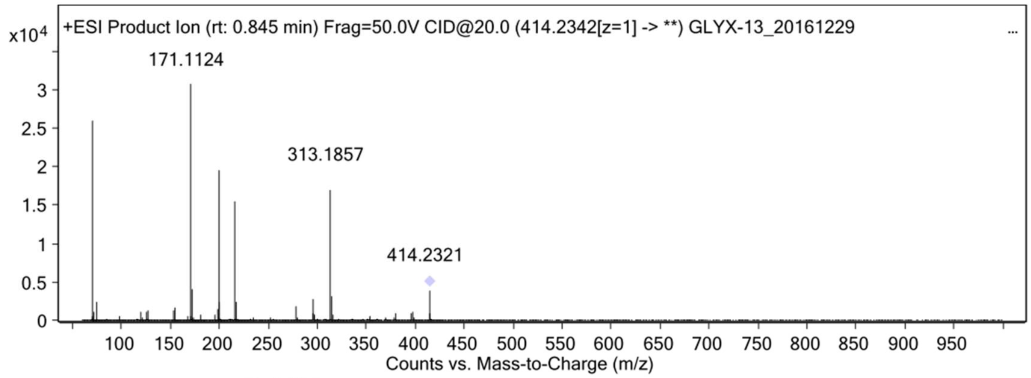

Peak List

m/z	z	Abund	Formula	Ion
70.0651	1	25984.63	C4 H8 N	M+
74.0606	1	2489.94	C3 H8 N O	M+
171.1124	1	30735.9		
172.1157	1	3996.63		
199.1071	1	19504.09		
216.134	1	15463		
296.1613	1	2725.16		
313.1857	1	17010.49		
314.1886	1	3202.7		
414.2321	1	3960.93		

Figure S20. MS/MS spectra of GLYX-13

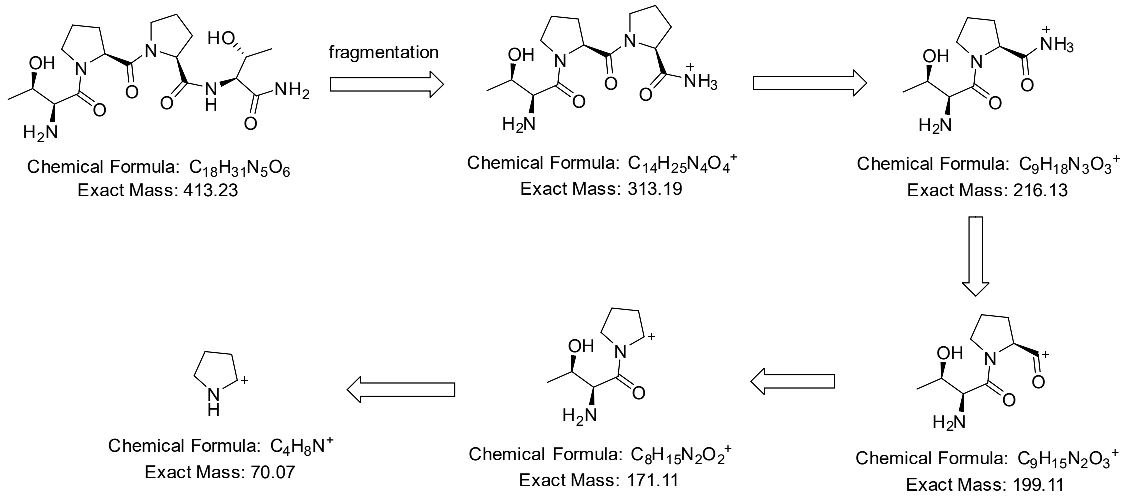

Figure S21. MS/MS fragment ion analysis of GLYX-13
